# Recommendations for maternal mental health policy in India

**DOI:** 10.1057/s41271-022-00384-4

**Published:** 2023-01-09

**Authors:** Urvashi Priyadarshini, Arathi P. Rao, Sambit Dash

**Affiliations:** 1 Bengaluru, India; 2 Department of Health Policy, Prasanna School of Public Health, Manipal Academy of Higher Education, Manipal, India; 3 Division of Biochemistry, Department of Basic Medical Sciences, Manipal Academy of Higher Education, Manipal, Karnataka India

**Keywords:** Perinatal mental health, Maternal mental health, Policy recommendation, Policy brief

## Abstract

Mental illnesses are a serious concern in India where every seventh person suffers from mental health problems—with women more affected than men. While the burden of perinatal mental illnesses grows, India lacks exclusive policies to address it. The COVID-19 pandemic has had an impact on routine antenatal care and institutional deliveries and has also affected the mental health of pregnant women and mothers. We evaluated existing policies. Policy options were evaluated against criteria like cost–benefit analysis, administrative feasibility, human resources, and equity along with the intended and unintended consequences. We propose three policy options: (1) strengthening and focused implementation of the existing national mental health program (NMHP), (2) integrating mental health in the ongoing Reproductive, Maternal, Newborn, Child and Adolescent Health Program, and (3) including a ‘maternal’ component in NMHP. We offered policy recommendations to fill the gap in addressing the maternal mental health challenge in India.

## Key messages

- The article prompts policymakers to take cognizance of the increasing burden of perinatal mental health problems in India.
- The National Mental Health Program is still underdeveloped in many parts of India.
- The proposed recommendations help draw a comprehensive policy for addressing maternal mental health.

## Introduction

Research has indicated that India has a high burden of mental health disorders. According to the India State-Level Disease Burden Initiative, in 2017, approximately, 197.3 million Indians suffered from mental illnesses, meaning every seventh Indian was affected by mental illnesses of varying severity [1]. The study also estimated the percentage prevalence of depressive and anxiety disorders to be higher in Indian women (3.9%) than men (2.7%) [1].

Pregnancy and the postnatal period are psychologically distressing times for women due to physiological changes. Women experiencing familial conflict, abuse, financial constraints, or complications during pregnancy are especially at-risk for developing perinatal depression. In 2005, researchers estimated prevalence of antenatal and postnatal depressive disorders in high-income countries, to be 11% and 15%, respectively [2]. Some studies in low-and middle-income countries (LMICs) have reported estimated prevalence to be even higher, 15.6% antenatally and 19.8% postnatally [3]. Despite such high prevalence, maternal mental health problems remain “under-recognized and under-treated” [4]. In India, recent studies estimated the prevalence of perinatal depression to be between 14 and 24% [5–7]. Researchers estimated in 2020 the magnitude of other pregnancy-related disorders such as pregnancy-related stress and anxiety to be 30.9% [8] and 23% [9], respectively.

The reproductive, maternal, newborn, child and adolescent health (RMNCH + A) Program, in effect since 2013, caters to pregnancy and childbirth-related health needs [10]. Its focus is limited to the physical well-being of the woman-childbearing or mother [10]. The mental health needs of the Indian population fall under the national mental health program (NMHP) of the non-communicable disease control program [11]. Even after several revisions of the program through five-year plans, it still fails to encompass maternal mental health. India also launched a National Mental Health Policy in 2014 focused on vulnerable populations and de-stigmatization of mental health across India’s vast population, but it too fails to include women in the perinatal period as a population susceptible to psychiatric problems [12].

## Underlying factors

Despite well-planned measures delineated in the NMHP’s 5-year plan and relevant policies, the treatment or management of general psychiatric illnesses, including maternal mental health, is largely unavailable or of inferior quality throughout the country.

### Lack of awareness, mental health literacy, and social stigma

Gaiha et al*.* revealed that a significant population in India remains unaware of mental illnesses and the availability of mental health care [13]. Socio-cultural factors influenced this study population to view mental illnesses as “evil spirits,” the influence of “black magic,” or caused by sins of a past life [13]. Thus, lack of knowledge about mental illnesses also hindered patients from seeking appropriate care. Persons suffering from mental illnesses face stigma and discrimination from families and society; this makes them reluctant to seek professional help [12]. For people suffering from mental illnesses, a study conducted by Gaiha et al. showed negative impact in relationships and social contact. [13]. India’s NMHP addresses de-stigmatization of mental illness as a primary objective to achieve through information, education, and communication activities. It works with the district mental health program (DMHP), an administrative unit that implements the NMHP in the state [11]. However, the national mental health survey (NMHS) of 2015–2016 demonstrated that the current mental health education activities were “isolated, sporadic and invisible” and “lacked focus and direction” [14].

### Financing

For 2021–2022, India’s Ministry of Finance allotted to the Ministry of Health and Family Welfare Rs. 712,690 million, about 3% of India’s Gross Domestic Product [15]. Of this, the government dedicated a total of Rs. 5970 million to mental health care and only Rs. 400 million to NMHP [15]. Most funds supported two centrally controlled institutions: Rs. 5000 million to the National Institute of Mental Health and Sciences (NIMHANS) in Bengaluru and Rs. 570 million to Lokpriya Gopinath Bordoloi Regional Institute of Mental Health in Tezpur [15]. The institutes directed these funds to mental health research and surveys such as the NMHS. Funding for the NMHP remained unchanged from the previous year. According to the NMHS, most of the budget for mental health supported salaries and pharmaceuticals [14]. Program officials lacked clarity about the responsibilities of the Centre versus those of the State. Only the states of Kerala and Gujarat had a ‘budget head’ for mental health. The survey also found that only 1% of the total health budget for the state was available for mental health. Some states reported that they “were unable to use the available funds” due to a lack of “clear mechanisms and guidelines.”

### Health information system

The 2015–2016 NMHS survey showed all the states included to be in different stages of implementing their health management information systems [14]. Only five states (Chhattisgarh, Gujarat, Madhya Pradesh, and Punjab) included mental health routinely in these information systems. Where the system was in place, it recorded information only for patients registered for treatment of psychosis, neurosis, mental retardation, and epilepsy. The system focused primarily on maternal and child health followed by communicable diseases and other national programs. In several states, the health management information system needs to better document cases involving mental health. States also need to upgrade it to include mental health illnesses, including maternal mental health.

### Implementation

According to the 2015–2016 NMHS, all states agreed on a need for an action plan that would include activities for implementation, budget, and financing; responsible agencies and designated individuals; indicators for monitoring and evaluation; and clearly define expected outcomes approved by state authorities [14]. The DMHP, the implementation arm of the NMHP, had limited reach; only one-third of Indian states reached more than 50% of their populations with DMHP services. The percentage of districts covered under DMHP varied by state, from 13.4 in Punjab to 100% in Kerala. The survey showed implementation of monitoring and evaluation of the NMHP to have been the most neglected domain [14]. Only Tamil Nadu and Gujarat reported a periodic or methodical monitoring system [14]. The survey also showed coordination among the Centre, states, districts, departments, institutions, and peripheral agencies to have been functionally absent, leading to delays in implementation [14].

### Human resources

Healthcare professionals in India are scarce, especially in mental health. Psychiatrists, clinical psychologists, and psychiatric social workers practice mainly in urban areas; limited availability remains a barrier to mental health care for all [14]. According to NMHS 2015–2016, the number of psychiatrists in all states, except Kerala, was fewer than the requirement of one psychiatrist per 100,000 population, ranging from the lowest of 0.05 in Madhya Pradesh, to 0.3 in Assam, 0.6 in West Bengal, and 1.2 in Kerala, the highest [14].

### Infrastructure

Healthcare facilities dedicated to mental health are few. All states evaluated in the 2015–2016 NMHS, except Manipur, had at least one mental hospital; all states had medical colleges with psychiatric departments, and general hospitals with psychiatric units. A few other states had also had de-addiction centers [14]. Combined statistics in all surveyed states 2015–2016 showed 450 mobile mental health units and 249 de-addiction centers [14]. Human resources and facilities remain insufficient to meet the mental health care needs, with variations of services among states, and uneven distribution [14]. Little information is available about private mental health care institutions [14].

### Legislation

According to the 2015–2016 NMHS, implementation of mental health legislation varied across states with no formal or informal evaluation reports available for many individual states [14]. Implementation was most robust in Kerala, Punjab, and Gujarat [14]. In 2017, India implemented the Mental Healthcare Act to ensure the availability of mental healthcare for all along with decriminalizing suicide and promoting their rehabilitation into society; however, the extent of implementation is yet to be evaluated [16, 17]. To combat the rising prevalence of mental illnesses in India, we analyzed mental and reproductive health programs as a basis to recommend policies for integrating maternal mental health services into primary healthcare.

## Evaluation

All healthcare programs and policies in India fall under the umbrella of the National Health Mission that operates under the Ministry of Health and Family Welfare. We conducted a comprehensive search for programs focused on mental and reproductive health. We analyzed all programs in detail and offer recommendations to improve maternal mental health. We also provided a justification for each option. Across India the health situation differs from state to state. By providing options, we hope policymakers will embrace those best suited to each situation. We structured the policy options around two programs: the NMHP and the Reproductive, Maternal, Neonatal, Child, and Adolescent Health Program (RMNCH + A). We also suggested an implementation strategy in accord with current infrastructure and human resources.

We evaluated the policy options using criteria related to cost–benefit or cost-effectiveness, administrative feasibility, human resources, sustainability, legal provisions, and equity. We also addressed barriers to implementation.

### Policy options recommendations

Below we offer three policy options to facilitate formulation and execution of a comprehensive policy that is administratively feasible, cost-effective, socially acceptable, and culturally appropriate.

#### Policy option 1: better implementation of the existing NMHP

When India launched NMHP in 1982, a key strategy was to integrate mental health with primary healthcare. The initial model lacked clarity, and inadequate funding to lack of funding, skilled human resources, and “managerial skill at the community level” undermined its sustainability. The Ministry of Health and Family Welfare revised the program repeatedly in the process introducing the DMHP [18]. Revisions targeted the most vulnerable and underprivileged populations [18] with objectives to decrease distress, disability, premature mortality associated with mental illness, and enhance recovery from mental illnesses. Other objectives included reducing stigma, promoting community participation, increasing access to mental health care services, ensuring the rights of persons with mental illness, integrating MH with other programs such as rural and child health, staff motivating and empowering them in the workplace, improving infrastructure for mental health service delivery, generating knowledge and evidence for service delivery, and establishing governance, administrative, and accountability mechanisms [18].

The goals, objectives, and strategies under the NMHP and DMHP seem holistic but implementation has been weak (Table 1). If the DMHP is strengthened in all districts and all recommendations of the NMHS were implemented, the NMHP could provide adequate care for maternal mental health and for all other persons with mental illness.

**Table 1:** Evaluation of policy option 1

Option 1
Cost benefit analysis
A cost-effective option as the mechanisms for policy execution are already in place
Additional finances may be required for strengthening of DMHP, implementation of DMHP (where hasn’t been implemented yet), recruitment and training of required staff, and information, education, and communication activities
Administrative feasibility
States must increase compliance with policies and strategies and update the health management information systems for program monitoring and evaluation
Because many districts have already implemented the program in some way, complete implementation should be feasible
Facilities will need to prepare detailed treatment or management plans because many pharmaceuticals and treatment procedures are contraindicated in pregnancy
Human resources
Facilities run by health ministries at State level Ministries of Health will recruit staff able to manage the population assigned to each healthcare establishment
Appropriate training at primary health center level will be needed to fulfill responsibilities and sensitization
Trained community health workers may be needed to supplement the teams of professionals for engaging the community and addressing stigma around mental illnesses
Sustainability
This policy option will be sustainable if authorities allocate sufficient budgets for strengthening program components and updating health management information systems to ensure evaluation of all components to support improvements

#### Policy option 2: integration of the mental health component in RMNCH + A

From the inception of the reproductive and child health program in 1997 to RMNCH + A, the Government of India successfully established a system of healthcare for women to safely manage their reproductive health, pregnancy, and childbirth. Government also launched cash-benefit schemes to ensure that pregnant women would attend their antenatal care visits regularly and prefer institutional delivery over home births. Community health workers such as accredited social health activists, auxiliary nurse midwives, and healthcare workers in primary healthcare centers and community healthcare centers function well as reflected across the country by dwindling rates of maternal and infant mortality. Cash benefits for four antenatal care visits, two postnatal care visits, and institutional delivery offer ‘windows of opportunity’ for the healthcare workers to screen women for perinatal mental illnesses (Table 2).

**Table 2:** Evaluation of policy option 2

Option 2
Cost benefit analysis
A new mental health component may incur additional costs to finance training non-specialist staff, establish referral channels, recruit additional mental health professionals, pharmaceuticals, equipment, and infrastructure
Administrative feasibility
A thorough revision of the RMNCH + A guidelines to include screening procedures, establish of referral channels and training procedures for healthcare providers
A detailed treatment or management plan involving maternal healthcare delivery units, including drugs and procedures safe for pregnancy
Human resources
Addition of a mental health component to RMNCH + A may require recruitment of additional staff
Training for non-specialist staff will be essential to screen for maternal mental health disorders and make referrals when needed. Training will be needed to fulfill responsibilities and sensitization of communities on mental health issues
Trained community health workers may be added to the team of professionals to engage the community and address stigma around mental health illnesses
Sustainability
This policy option will be sustainable if health care workers are trained, and if adequate finances are available for program activities and measures, such as updating health management information systems to ensure evaluation of all components to support improvements and sustain the program

Because the community health care workers are well acquainted with the pregnant women and mothers who hail from the same communities where the workers made door-to-door visits, the women may be more receptive to interventions provided under new policy. South Africa implemented a similar intervention called the Perinatal Mental Health Project that included mental health services for perinatal women in limited resource settings [19].

#### Policy option 3: inclusion of a “maternal” component in the NMHP

To avoid over-burdening the health care workers involved with the functioning of the RMNCH + A program, a third option could be integration of a dedicated ‘maternal’ component in the NMHP. Thirty-five years of operating the NMHP has generated plans and resources to support addition of a new domain. Successful implementation of the NMHP in some regions, such as the Thiruvananthapuram district in Kerala, demonstrates feasibility. Implementation of DMHP began in 1999 with formation of a district mental health team followed by training of health care workers, and sensitization of communities on mental health issues [19]. The multidisciplinary team provided outreach services, managed complicated cases, and supported community health workers [20]. The district-maintained record books (offline data) for patient records, used available funds, and performed program activities [19]. Availability of psychotropic drugs for treatment proved to be a key feature [19]. All health facilities (including district hospitals, primary, and community healthcare centers) followed standard procurement program procedures (based on guidelines), and according to a requirement raised by the program officials, Government made timely availability of these drugs [20]. The World Health Organization hailed this implementation as a success [20].

Because the burden of maternal mental illnesses is high in the country and has an intergenerational impact, an independent component under the umbrella of the NMHP may prove effective for women who are in dire need of attention of the sort described just above (Table 3).

**Table 3:** Evaluation of policy option 3

Option 3
Cost benefit analysis
Additional costs will include funding to strengthen the existing program and its implementation and to establish a new ‘maternal’ component requiring recruitment of staff, training, and procurement of suitable pharmaceuticals
Administrative feasibility
This may be a time-consuming alternative as it requires improving the existing program before establishing maternal units with additional drug lists and safe procedures approved for pregnant women
Human resources
Recruitment of health care workers specializing in maternal care and additional human resources to manage the (expected) surge in patients will be required to sustain a maternal component in the mental health program
Sustainability
This policy option will be sustainable if budget allocation is adequate. It will be necessary to strengthen program components including reporting systems and recruitment of trained health care workers and staff

India’s government designed both the RMNCH + A and NMHP to cater to rural as well as urban populations. Thus, we expect both can maintain equity in quality and quantity of services. Because healthcare is often inadequate in rural areas, extra measures will be needed, including periodic monitoring and evaluation to assess the quality of services provided. All three policy options meet the legal requirements of the Mental Healthcare Act of 2017. Even so, success will depend on popular acceptance of the reality of mental health illnesses and their cure through community sensitization.

## Implementation

We recommend starting with a pilot project before nationwide implementation of any option, including three elements:

### Comprehensive plan for all levels

Even though only a few districts will pilot the new approaches, comprehensive planning is needed to address the needs of both urban and rural populations. Planning should include establishing reporting channels, setting up a system for accountability, and for monitoring and evaluating the program. Funds need to be earmarked for training, information, education, and communication activities, procurement of drugs, improvement of infrastructure, salaries, among other program-related activities.

### Social mobilization

To generate awareness to address social stigma affecting mental health, and maternal mental health, mechanisms are needed to disseminate communication strategies. Information, education, and communication campaigns by community health workers and community leaders will also be essential to sensitize and educate the vast population about the importance of mental health, why and how to seek help, and about mental health services offered and their benefits.

### Human resource

Increasing hiring of health professionals specializing in mental health care will be essential to establish and carry out treatment and management protocols, including use of safe psychotropic drugs for use in childbearing and breastfeeding women, for institutional care, and psychosocial interventions to limit reliance on institutional care.

### Intended and unintended consequences of policy options

We have attempted to evaluate consequences, intended or not, of each policy alternative to facilitate responses that may be needed (Table 4).

**Table 4:** Intended and unintended consequences of policy options

Option 1	Option 2	Option 3
Intended consequences		
Reduced maternal and infant morbidity and mortality rates attributable to poor mental health Increased employment rates due to staff recruitment Increased MH literacy in the population		
Unintended consequences		
	Avoidance of RMNCH + A services due to continued prejudice against mental illness	
Discrimination against advocates of mental health Due to increased allocation of funds from other programs or activities, reductions elsewhere may affect the program-related activities Increased staff responsibilities may lead to burnout		

### Barriers to implementation

Factors that may hamper effective integration of maternal mental health services into primary care may include continued lack of public awareness and social stigma. Resources needed, such as the upgrading infrastructure and stocking of appropriate drugs, may not be cost-effective. A shortage of human resources or trained personnel may interfere due to lack of new recruitment, disinterest in mental health services, or poor working conditions. Funds allotted could be inadequate.

## Conclusions

Maternal mental disorders not only impact the mother but have a significant effect on the child and family. Stigma and other social barriers inhibit women from seeking needed help. Uncertainty produced by the ongoing COVID-19 pandemic, likely to exacerbate mental health issues, will require all involved to acknowledge and address this complicating factor [21]. Also, inadequate availability of medical care and fear of infection predispose pregnant women and mothers to increased risk of developing mental illnesses. Because maternal mental health directly impacts maternal morbidity and mortality with an intergenerational health impact on children, improving the maternal mental health of the Indian population will be needed for the country to achieve its health targets for women and children. Doing so will be an important step toward achieving the Sustainable Developmental Goals (SDG 3.4). India should also follow the example of many developed and developing countries (Canada, Australia, and South Africa, to name a few) that are in the process of implementing their policies for women in the perinatal period, by integrating maternal mental health care with primary care.
